# Adolescent suicide assessment and management in primary care

**DOI:** 10.1186/s12887-022-03454-4

**Published:** 2022-07-02

**Authors:** M. Aalsma, J. Keys, S. Ferrin, M. Shan, T. Garbuz, T. Scott, Z. Adams, L. Hulvershorn, S. Downs

**Affiliations:** 1 Division of Adolescent Medicine, Adolescent Behavioral Health Research Program, Department of Pediatrics, Indiana University School of Medicine, 410 W. 10th St. Rm 2025, Indianapolis, IN USA; 2 General Pediatrics and Adolescent Medicine, Department of Pediatrics, University of North, Carolina School of Medicine, Chapel Hill, NC USA; 3 Department of Biostatistics, School of Public Health, Indiana University School of Medicine, Indianapolis, IN USA; 4 Department of Psychiatry, Adolescent Behavioral Health Research Program, Indiana University School of Medicine, Indianapolis, IN USA; 5 Department of Pediatrics and Center for Biomedical Informatics, Wake Forest School of Medicine, Winston-Salem, NC USA

**Keywords:** Adolescent, Suicide, Screening, Primary care, Computer decision support systems

## Abstract

**Background:**

To understand how suicide management occurs within the primary care setting in terms of follow-up assessments and referral practices.

**Methods:**

At an initial primary care visit, adolescents (aged 12–20 years old) completed electronic screening. Data were focused on youth who endorsed a suicidal risk item while completing screening at two Midwestern primary care clinics. Data were collected through retrospective chart reviews to analyze actions taken by the primary care physician at the youth’s initial visit and follow-up visit within the next 12 months.

**Results:**

At initial visits 200 adolescents endorsed a suicidal risk item and 39 (19.5%) were considered to be concerning by their primary care physician. The average age was 14.7 years old (SD ± 2.0). Seventy-two percent (*n* = 144) were female, and 65% (*n* = 129) identified as Black. At initial visits, significant differences between suicidal concern groups were found in reporting active suicidal ideation, past suicide attempts, those who were referred to behavioral health counseling, and those who had a diagnosis of depression. Interestingly, only 13% (*n* = 25) of all patients who endorsed the suicide item were asked whether or not there were weapons in their home and primary care providers asked only 7% (*n* = 13) of all patients whether they had a safety plan.

**Conclusions:**

There was inconsistent follow-up for adolescents with a history of suicide concerns. At this time, national guidelines do not exist regarding primary care follow-up of youth with suicide concerns. Guidelines are a necessary precursor for practice improvement.

**Trial Registration:**

Clinical Trials Registry: NCT02244138. Registration date, September 1, 2014.

**Supplementary Information:**

The online version contains supplementary material available at 10.1186/s12887-022-03454-4.

## Introduction

Suicide was the second leading cause of death for persons aged 10–14 years old in the United States in 2020 [1]. Suicidal ideation is relatively common among adolescents in the US with 19% of adolescents aged 13–18 years old experiencing suicidal ideation (*having seriously thought about completing suicide) *[2]. Suicidal ideation increases an adolescents likelihood of subsequent planning and thus attempting suicide, even though more than 80% of adolescents that have attempted suicide have already received some type of mental health treatment [3].

One method to identify adolescents at risk for suicide is preventative screening for suicidal thoughts and behaviors within the primary care setting [4]. This is important as 50% or more of adolescents attend a primary visit each year for any health related reason [5]. It is common for those who die by suicide to use primary care facilities within the year prior to suicide. For instance, in a study of primarily adults that died by suicide, 50% had attended a medical visit within the 4 weeks prior to death [6].

Standards for management suicide risk are not clear in the extant research literature. The Guidelines for Adolescent Depression in Primary Care (GLAD-PC) toolkit recommends initial management and safety planning, to include restricting access to lethal means, preparing the family to monitor suicide risk, establishing emergency contacts and crisis resources, participating in mental health treatment, and arranging follow-up care [7, 8]. Providers are encouraged to refer suicidal youth to crisis or emergency services and inpatient care, if necessary. Similarly, the Suicide Prevention Resource Center (SPRC) toolkit provides protocol for safety planning, referral to evidence-based treatment with mental health professionals, hospitalization for high risk adolescents, and close follow-up to reduce suicide risk [9]. Other safety guidelines differ based on severity of suicide risk and the adolescent’s needs. Interventions for adolescents at moderate or high risk include immediate evaluation by a mental health professional whereas low risk individuals need appropriate follow-up and/or referral for evalulation [10]. However, on-going primary care management of youth with suicidal intent and behaviors are unclear.

Few studies describe the follow-up care for adolescents who appear at primary care settings with a positive risk for suicide. To our knowledge, the largest cohort study that monitored the follow-up care for adolescents who had a recorded event of self-harm, occurred in the United Kingdom in 2017. Self-harm was defined as any act of self-poisoning or self-injury, regardless of motivation, which includes non-suicidal self-injury as well as suicide attempts [11]. Among young people in the study who had a record of self-harm, a diagnosis of depression was highly prevalent (recorded for over a third of the young women and over a quarter of the young men). The majority (55%) did not have a referral for mental health services in their primary care record up to 12 months following the initial self-harm incident; and 20% were prescribed antidepressants during the same time period [12]. A more recent study of adolescents in primary care found 34% of youth had a referral for mental health services by their pediatrician [13].

Although primary care providers are not tasked with treating youth with suicidal behaviors, the reality is primary care providers are increasingly managing psychiatric treatment for their patients. Recent estimates indicate that roughly one-third of patients were being treated for mental health by their primary care provider [14]. Moreover, although visits to psychiatrists have remained fairly stable, psychiatric medication visits to primary care have increased substantially [15]. In sum, primary care providers are managing a surging population of youth with mental health problems that are complex and include the management of psychiatric medication, referral for treatment, and the ongoing assessment of symptoms and medications. In addition, youth present to primary care with suicidal behavior which is a complex bio-psycho-social phenomenon. Very few guidelines exist to inform this important work.

The overall purpose of this project is to describe suicide management in the primary care setting, including follow-up assessments and referral practices, within the 12-month period following the first identification of suicidal risk. Our study expands on this knowledge, by recording physicians’ determinations of individual patients’ suicidal intent following a youth’s indication for suicide risk on an electronic screener during a primary care visit. This study focuses on the long term follow-up of youth determined to be of concern for suicidality by the physician, which was recorded as *no concern* or *suicidal concern* in the electronic screening tool.

## Methods

### Study design

This study consisted of a descriptive, retrospective chart review for 200 adolescent patients (between 12–20 year) who screened positive for suicide risk. All charts with a positive indication of suicide risk (i.e. youth marked yes to electronic screening question of suicide risk, see below) were reviewed regardless of providers designation of need for follow-up care by the primary care provider. This designation of need for follow-up care was based on the physician indicating if there was a “concern” versus “no concern” for youth that endorsed the suicide risk item.

### Participants and settings

Participants were identified through a Computer Decision Support System (CDSS), the Child Health Improvement through Computer Automation (CHICA) system. The CHICA system was implemented by the research team in two primary care clinics in an urban Midwestern setting. One component of the CHICA system is a Pre-Screener Form (PSF), which is administered to patients via tablet upon check-in to their primary care visit. For adolescents, the PSF consisted of a 20-item questionnaire, including screening items for depression, substance use, diet, and sexual behaviors. Adolescents were specifically screened for suicide risk based on the American Academy of Pediatrics and American Medical Associations recommendations: “Have you ever seriously thought about killing yourself, made a plan, or actually tried to kill yourself?” [10, 16, 17] The PSF was administered to adolescents between the ages of 12 and 20 years old. Based on patients’ responses to these questions, a physician worksheet is generated prioritizing the top six health needs of the patient with physician action prompts based on Bright Future guidelines. Detailed descriptions of the CHICA system have been published elsewhere [18–21].

The primary care clinics where these visits took place were part of the local county hospital, and primary care providers at these clinics were trained in pediatrics with some care provided by physicians board-certified in adolescent medicine. The team received IRB approval to conduct this study from the Indiana University. The IRB approval included an approval for the experimental pediatric clinic waitlist control condition as well as a waiver of individual consent/assent of study participants. All study procedures were conducted in accordance with the Indiana University IRB guidelines and procedures.

### Chart abstraction

Retrospective chart reviews were conducted on all patients screening positive for risk of suicide at their initial primary care visit from 2014–2017 (*n* = 200 patients). The primary care notes for each primary care visit during this time frame were reviewed, and data related to the variables listed in Table 1 were entered into a spreadsheet. Variables chosen for analysis were based on 4 broad areas. The first group included patient characteristics, the second group of variables were focused on suicide and self-harm behavior. Given the importance of behavioral health care for individuals with suicidality, the final 2 groups of variables assessed behavioral health care and psychiatric medications. The unit of analysis was primary care visit, thus, individuals could contribute multiple visits to the dataset.

**Table 1:** Variables from medical record

Variable Label	Description	Response Scale
**Basic Visit Information**		
Sex	Sex of the patient	Numeric score
Age at incident visit	Provider reported the age of the patient at the visit	Free Response
Race	Provider reported the race of the patient at the visit	Free Response
**Suicidal Ideation/Attempts & Self-Harm**		
Active suicide ideation	Provider reported if patient had active suicidal ideation	Yes/No
Past suicide ideation	Provider reported if patient had a history of suicidal ideation	Yes/No
Past suicide attempt(s)	Provider reported if patient had past suicide attempt(s)	Yes/No
Suicide attempt history	Provider reported details regarding patient’s past suicide attempt(s)	Free Response
Weapons	Provider reported asking if patient had any weapons at home	Yes/No
Safety plan	Provider reported asking patient about safety plan	Yes/No
**Behavioral Health**		
Mental health referral	Provider referred patient to outpatient mental health services	Numeric score
Co-morbid diagnoses	Behavioral health-related diagnoses listed by the provider	Free Response
Hospitalizations^a^	Provider reported if patient had been in an inpatient psychiatric setting since previous visit	Numeric score
Visit engagement^a^	Provider reported if patient has attended behavioral health visits	Numeric score
**Psychiatric Medication**		
Psychiatric medications	Provider reported starting patient on psychiatric medication	Yes/No
Psychiatric medication list	List of psychiatric medications prescribed to patient	Free response
Psychiatric medication continuity^a^	Provider reported if patient continued to take prescribed psych meds	Numeric score

^a^Coded only at follow-up visit

### Data analysis

Twenty percent of charts were reviewed by two reviewers to check interrater reliability. The pooled kappa was 0.54, indicating moderate agreement. Patient characteristics and records at incident visits were summarized as frequency and percent for categorical variables, and as mean and standard deviation for continuous variables. For patients who had follow-up visits, we summarized their assessment and referral practices, change of medication and change of symptom status in the follow-up visits by suicide risk levels. Risk level included when the provider determined youth was at risk for suicide (suicide concern) and not at risk for suicide (no concern). Univariate differences in patient records between suicide risk levels were compared using the Student t-test or Wilcoxon test for continuous variables and the chi-square test for categorical variables. Patient race was regrouped as “black” and “non-black” due to small samples in some of the categories. To limit the risk of reidentification (i.e. individuals providing anonymous data being de-anonymized and identified), only cell sizes of 10 or more participants are listed. All analyses were conducted using SAS 9.4 (SAS Institute Inc., 2013) with a 5% significance level.

## Results

### Description of initial visit for suicide risk management

Among the 200 patients endorsing the suicidal risk item, the primary care physician considered 19.5% (*n* = 39) to represent a suicidal concern after further assessment. Rates of suicide management during the initial primary care visit are presented in Supplemental Table 1. The average age of all 200 patients at the first positive suicide endorsement was 14.7 years old (SD ± 2.0). Seventy-two percent (*n* = 144) were female, and 65% (*n* = 129) identified as Black. Between those the physician considered a suicidal concern (*n* = 39) and the no concern group (*n* = 161), there was a significant difference in reported active suicidal ideation, higher in the suicide concern group (Cell size not displayed as they are below 10 youth; *p*-value < 0.0001). The suicidal concern group reported past sucide ideation significantly more often than the no concern group [suicidal concern = 26 (67%); no concern = 61 (38%); *p*-value < 0.001]. The suicide concern group also reported significantly higher rates of past suicide attempts (Cell size not displayed as they are below 10 youth; *p*-value < 0.0001].

Records indicated that 87% (*n* = 173) of patients had a mental health history and/or status documented in their visit note. Thirteen percent (*n* = 25) of patients who endorsed the suicide item were asked whether or not there were weapons in their home. Primary care providers asked 7% (*n* = 13) of patients whether they had a safety plan, and referred 51% (*n* = 102) to behavioral health treatment. The percentage of patients who had been referred to behavioral health counseling was higher in the suicidal concern group versus the no concern group [suicidal concern = 28 (72%); no concern = 74 (46%); *p*-value < 0.003].

At the index visit when suicide screening occurred in primary care, 56% (*n* = 112) of patients had a diagnosis of depression, with the suicidal concern group having a slightly higher percentage when compared with the no concern group [suicidal concern = 28 (72%); no concern = 84 (52%); *p*-value < 0.02]. Differences in diagnoses of anxiety, autism spectrum disorder, and other mental health ailments were not significant between suicidal concern and no concern groups. Primary care providers started 35% (*n* = 69) of patients on psychotropic medications. Psychotropic medication type (antidepressant, antianxiety, etc.) was not statistically significantly different between suicide concern and no concern groups (*p* values ranged from 0.07 – 0.97, depending on medication).

### Description of follow-up care for suicide risk management

Of the 200 patients endorsing the suicidal risk item at their initial visit, 70.5% (*n* = 141) had a follow-up primary care based visit within the next 12 months. Rates of suicide risk management during the one-year follow-up period are presented in Supplemental Table 2. At follow-up, the rates of youth with active suicidal ideation, reported past suicidal ideation, and reported past suicide attempts were not statistically significantly different.

Records indicated that 80% (*n* = 113) of the 141 patients who had follow-up had a documented mental health history and/or status at their follow-up visit. No significant differences were noted between suicide risk levels [suicidal concern = 21 (75%); no concern = 92 (81%); *p*-value = 0.44]. Twelve patients who attended a primary care visit during the follow-up period were asked about weapons at home, with no significant difference between suicide risk levels (*p*-value = 0.77). Primary care providers also asked patients whether they had a safety plan, with no significant difference between suicide risk levels [*p*-value = 0.59].

Between the suicidal concern and no concern groups, there was no difference in how often patients were referred to behavioral health counseling [suicidal concern = 14 (50%); no concern = 54 (48%); *p*-value = 0.83], and 26% (*n* = 37) of patients were compliant with their behavioral health visits. During the year follow-up period, 15% (*n* = 21) of patients were hospitalized for unspecified reasons. There was no significant difference in hospitalization between groups. Psychotropic medication type was also not significantly different between suicide concern and no concerns groups (*p* values ranged from 0.06 – 0.98).

Table 2 displays the change in the number of patients on different medications from incident visit to follow up visits. There were no differences from incident to follow-up visit in the 3 most commonly prescribed psychiatric medications (antidepressant, atypical antipsychotic and stimulant medications).

**Table 2:** Follow-up medications

	**# of Patients at Incident Visit**	**% of Patients at Incident Visit**	**# of Patients at Follow-Up Visit**	**% of Patients at Follow-Up Visit**
Antidepressant	33	23%	35	25%
Atypical antipsychotic	14	10%	10	7%
Stimulant	19	14%	21	15%

## Discussion

After the identification of youths’ risk for suicidal behaviors or thoughts at the first primary care visit, we found that the follow-up care actions taken by physicians included a low rate of discussions concerning weapons in the home and safety planning. However, rates of discussing the patient’s mental health history/status and referrals to behavioral health counseling were high. This was true, however, only for the suicidal concern group. Follow-up actions by physicians when youth appeared at primary care in the following 12 months were similar to those found at the initial visit, but there was no difference between suicidal concern groups on the rate of referrals to behavioral health counseling. Overall, we found a low rate of engagement to behavioral health counseling visits.

### Follow-up care actions by primary care providers

At initial visits to primary care, few youth were asked about whether or not there were weapons at home or had a discussion with their physician about safety plans, 13% and 7% respectively. Similar rates were found at follow-up care visits. Only 12 patients of the 141 were asked about weapons at home, with no significant difference between suicide risk levels. Furthermore, only 8 patients were asked if they had a safety plan. Mental health symptoms, such as depression, can influence whether or not individuals attempt suicide [22]. However, access to firearms are a more direct and significant risk factor. In fact, males who use a firearm to attempt suicide have the highest fatality rate of all suicide attempters within the United States [23]. The American Academy of Pediatricians (AAP) states that the primary care physician should inquire about suicide plans and “whether there are firearms in the home” when patients have responded positively to having seriously thought about suicide [10]. A recent study found pediatricians report asking about firearms in the home [24]. Moreover, a recent study of adults in primary care setting found physicians rarely queried about firearm access [25]. In sum, our low reported rates of physicians asking about weapons in the home and safety planning are concerning, and in order to ascertain if adolescents are safe after leaving primary care, these topics should be more frequently discussed. This is particularly important as effective interventions for firearm storage exist, specifically provision of devices for safe firearm storage [26].

In comparison, rates of referral to behavioral health counseling was higher at both initial and follow-up care in comparison to rates of safety planning and discussing access to weapons. There has been speculation that physicians may more readily refer their patients with a risk for suicide to outside mental health care providers because primary care physicians can view the care of suicidal patients as an increased vulnerability to malpractice complaints if an adverse event occurs after meeting with patient [27]. This could explain why our study found the referral rate to behavioral health counseling higher than discussions between providers and patients on access to weapons within the household and safety plans [27]. Unfortunatly, only 26% of patients referred to behavioral health went to the referral.

The rate of prescribed medications continuity was also reportedly low during follow-up visits (32%, *n*= 45). Psychiaric medication continuity is a significant issue for adolescents. A wide range of reasons exist for the lack of continuity. Qualitative interviews on the experiences of children, adolescents, and their parents recorded reasons for not contuing a medication including unintended continuity, forgetting to take medicine, not filling prescriptions, and forgetting medications when travelling [28]. Medicaion continuity may be improved by individualized medication treatment plans with the patients and their families before they leave the primary care setting, so that families will have an idea of the challenges that they will face adhering to medication regimens and will be better prepared to face possible challenges.

### Primary-care based guidelines for suicide care and management

The American Academy of Pediatrics (AAP) has a published policy addressing suicide and suicide attempts in adolescents [10]. The guidelines are helpful in the initial assessment of youth for suicide in primary care. However, guidelines for follow-up management of youth with suicidal concerns are lacking. A recent review of suicide screening interventions in primary care emphasize the importantce of education, screening, management of depression, and further assessment of suicide risk [29]. Primary care models for suicide intervention have been assessed. Wintersteen trained primary care staff to implement direct but brief interventions with youth who present with a risk for suicide, but also on training physicians to refer patients to behavioral health counseling in an outpatient setting rather than directly to the emergency department in acute risk cases [30].

### Limitations

In this study we were limited by the solitary use of medical records for determining rates of treatment and follow-up in our population because treatment and follow-up could have occurred outside of the study’s primary care settings. We do not have access to those records. Furthermore, any actions taken by the primary care physician that were not recorded in the medical file are unknown to the research team and cannot be reported as occurring, limiting the scope of our knowledge. Moreover, physicians interpreted the terms “concern” versus “no concern” with their own discretion. With that said, physicians were provided a handout detailing the steps in how to assess for suicidal concerns and establish a safety plan. Future research should elucidate how physicians determine risk for suicidality. Finally, any actions taken by other health providers, such as social workers or mental health specialists, could not be included in this analysis regardless of whether those reports contained information about the suicide risk management of the patient. Our study only had access to the medical records kept by the physicians within the primary care setting included in our study.

In addition, our study has a limited generalizability due to our sample being largely Black and female. Also, all data on suicide risk were identified through our CDSS which was implemented in only two primary care clinics within the Eskenazi health network in Indianapolis, Indiana.

## Conclusions

Suicide risk among adolescents within the United States is identified as a public health problem. This has led to efforts to limit suicide, such as the Zero Suicide initiative [31]. Given the results from our study we believe that the identification and management of adolescents at risk is important within the primary care setting, and that continued follow-up of adolescents after a primary care visit when suicidal ideations were endorsed is also important. At this time, however, national guidelines for primary-care based follow-up care of suicidal youth are lacking. Thus, practice organizations are encouraged to consider how best to not only initially assess for suicidal thoughts and intervene at an initial primary care visit, but also how best to follow-up with youth presenting with significant suicidal ideation.

## Supplementary Information

**Additional file 1: Supplemental Table 1.** Description of Initial Visit for Suicide Risk Management. **Supplemental Table 2.** Description of Follow-Up Care for SuicideRisk Management.

## Data Availability

The datasets used and/or analyzed during the current study are available from the corresponding author on reasonable request.

## Abbreviations

CDSS: Computer Decision Support System
CHICA: The Child Health Improvement through Computer Automation
PSF: Pre-Screener Form
