# Supplementary material for: Adolescent suicide assessment and management in primary care
Source: BMC Pediatr. 2022 Jul 2;22:389. doi: 10.1186/s12887-022-03454-4 (PMC9250265; doi:10.1186/s12887-022-03454-4)
Supplement: Supplementary file 1 — Additional file 1: Supplemental Table 1. Description of Initial Visit for Suicide Risk Management. Supplemental Table 2. Description of Follow-Up Care for SuicideRisk Management. [file 12887_2022_3454_MOESM1_ESM.docx]

**Supplemental Table 1. *Description of Initial Visit for Suicide Risk Management***

|  | **All youth  n=200** | | **No Concern n=161** | | **Suicidal Concern  n=39** | | | **P-Value** |
| --- | --- | --- | --- | --- | --- | --- | --- | --- |
|  | **N** | **%** | **N** | **%** | **N** | | **%** |  |
| **Basic Visit Information** | | | | | | | | |
| **Sex** | | | | | | | | 0.44 |
| Female | 144 | 72% | NA |  | NA | |  |  |
| Male | 56 | 28% | NA |  | NA | |  |  |
| **Age at Incident visit** | 14.7 ± 2.0 | | 14.7 ± 2.0 | | 14.7 ± 2.1 | | |  |
| **Race** | | | | | | | | 0.05 |
| Black | 129 | 65% | 109 | 68% | 20 | | 51% |  |
| Non-Black | 71 | 36% | 52 | 32% | 19 | | 49% |  |
| **Suicidal Ideation/Attempts & Self-Harm** | | | | | | | | |
| **Active suicide ideation** | | | | | | | | <0.0001 |
| No | 179 | 90% | 153 | 95% | | 26 | 67% |  |
| Yes | 21 | 11% | NA |  | | NA |  |  |
| **Past suicide ideation** | | | | | | | | <0.001 |
| No | 113 | 57% | 100 | 62% | | 13 | 33% |  |
| Yes | 87 | 44% | 61 | 38% | | 26 | 67% |  |
| **Past suicide attempt(s)** | | | | | | | | <0.0001 |
| No | 183 | 92% | 155 | 96% | | 28 | 72% |  |
| Yes | 17 | 9% | NA |  | | NA |  |  |
| **Mental health documentation** | | | | | | | | 0.88 |
| No | 27 | 14% | NA |  | NA | |  |  |
| Yes | 173 | 87% | NA |  | NA | |  |  |
| **Weapons** | | | | | | | | 0.94 |
| No | 175 | 88% | NA |  | NA | |  |  |
| Yes | 25 | 13% | NA |  | NA | |  |  |
| **Safety plan** | | | | | | | | <.0001 |
| No | 187 | 94% | NA |  | NA | |  |  |
| Yes | 13 | 7% | NA |  | NA | |  |  |
| **Psychiatric medications started** | | | | | | | | 0.83 |
| No | 131 | 66% | 106 | 66% | 25 | | 64% |  |
| Yes | 69 | 35% | 55 | 34% | 14 | | 36% |  |
| **Behavioral Health** | | | | | | | | |
| **Mental health referral** | | | | | | | | <0.01 |
| No | 98 | 49% | 87 | 54% | 11 | | 28% |  |
| Yes | 102 | 51% | 74 | 46% | 28 | | 72% |  |
| **Depression diagnosis** | | | | | | | | 0.02 |
| No | 88 | 44% | 77 | 48% | 11 | | 28% |  |
| Yes | 112 | 56% | 84 | 52% | 28 | | 72% |  |

NA = Data not available due to low cell size below 10 participants.

Supplemental **Table 2. *Description of Follow-Up Care for Suicide Risk Management***

|  | **All youth  n=141** | | **No Concern n=113** | | **Suicidal Concern  n=28** | | **P-Value** |
| --- | --- | --- | --- | --- | --- | --- | --- |
|  | **N** | **%** | **N** | **%** | **N** | **%** |  |
| **Suicidal Ideation/Attempts & Self-Harm** | | | | | | | |
| **Active suicide ideation** | | | | | | | 0.40 |
| No | 131 | 93% | 106 | 94% | 25 | 89% |  |
| Yes | 10 | 7% | NA |  | NA |  |  |
| **Past suicide ideation** | | | | | | | 0.47 |
| No | 108 | 77% | 88 | 78% | 20 | 71% |  |
| Yes | 33 | 23% | NA |  | NA |  |  |
| **Past suicide attempt(s)** | | | | | | | 0.38 |
| No | 127 | 90% | 103 | 91% | 24 | 86% |  |
| Yes | 14 | 10% | NA |  | NA |  |  |
| **Mental health documentation** | | | | | | | 0.44 |
| No | 28 | 20% | NA |  | NA |  |  |
| Yes | 113 | 80% | NA |  | NA |  |  |
| **Weapons** | | | | | | | 0.77 |
| No | 129 | 91% | NA |  | NA |  |  |
| Yes | 12 | 9% | NA |  | NA |  |  |
| **Safety plan** | | | | | | | 0.59 |
| No | 133 | 94% | NA |  | NA |  |  |
| Yes | NA |  | NA |  | NA |  |  |
| **Behavioral Health** | | | | | | | |
| **Mental health referral** | | | | | | | 0.83 |
| No | 73 | 52% | 59 | 52% | 14 | 50% |  |
| Yes | 68 | 48% | 54 | 48% | 14 | 50% |  |
| **Hospitalizations** | | | | | | | 0.62 |
| No | 120 | 85% | NA |  | NA |  |  |
| Yes | 21 | 15% | NA |  | NA |  |  |
| **Visit engagement** | | | | | | | 0.75 |
| No | 104 | 74% | NA |  | NA |  |  |
| Yes | 37 | 26% | NA |  | NA |  |  |

NA = Data not available due to cell size below 10 participants.
